# Implementation of multiplex PCR diagnostics for gastrointestinal pathogens linked to increase of notified Shiga toxin-producing *Escherichia coli* cases in Norway, 2007–2017

**DOI:** 10.1007/s10096-019-03475-5

**Published:** 2019-01-24

**Authors:** Gaute Reier Jenssen, Lamprini Veneti, Heidi Lange, Line Vold, Umaer Naseer, Lin T. Brandal

**Affiliations:** 1 Department of Zoonotic, Food- and Waterborne Infections, Division for Infection Control and Environmental Health, Norwegian Institute of Public Health (NIPH), Postboks 4404, Nydalen, NO-0403 Oslo, Norway; 2 Faculty of Medicine, University of Oslo, Oslo, Norway; 3 Oslo University Hospital, Oslo, Norway

**Keywords:** STEC diagnostic, Multiplex PCR panels, Incidence of STEC, High-virulent STEC, Low-virulent STEC

## Abstract

The aim of this study was to investigate implementation of multiplex PCR assays (broad screening PCR) on the distribution and characteristics of notified Shiga toxin-producing *Escherichia coli* (STEC) cases in Norway, 2007–2017. We described STEC cases notified to the Norwegian Surveillance System for Communicable Diseases (MSIS), 2007–2017 and categorised cases as high-virulent, low-virulent or unclassifiable STEC infections based on guidelines for follow-up of STEC cases. We conducted descriptive analysis and time series analysis allowing for trends and seasonality, and calculated adjusted incidence rate ratios (aIRR) using negative binomial regression for laboratories with and without broad screening PCR. A total of 1458 STEC cases were notified to MSIS (2007–2017), median age 21 years, 51% female. Cases were categorised as having 475 (33%) high-virulent, 652 (45%) low-virulent, and 331 (23%) unclassifiable STEC infections. We observed a higher increasing monthly trend in cases (aIRR = 1.020; 95% CI 1.016–1.024) notified from laboratories with broad screening PCR (*n* = 4) compared to laboratories (*n* = 17) without (aIRR = 1.011; 95% CI 1.007–1.014). Notification of low-virulent STEC infections increased from laboratories with broad screening PCR. The increase in notified STEC cases was prominent in cases categorised with a low-virulent STEC infection and largely attributable to unselective screening methods. We recommend NIPH to maintain differentiated control measures for STEC cases to avoid follow-up of low-virulent STEC infections. We recommend microbiological laboratories in Norway to consider a more cost-effective broad screening PCR strategy that enables differentiation of high-virulent STEC infections.

**Electronic supplementary material:**

The online version of this article (10.1007/s10096-019-03475-5) contains supplementary material, which is available to authorized users.

## Introduction

Shiga toxin-producing *Escherichia coli* (STEC) infection may lead to mild gastroenteritis, haemorrhagic colitis or the life-threatening complication haemolytic-uraemic syndrome (HUS) [1]. An estimated 5–10% of patients with a STEC infection develop HUS, a number that may be higher when related to outbreaks [2]. Factors related to both the host and STEC have been associated with an increased risk for development of HUS. Young age, as well as the presence of Shiga toxin-producing gene *stx2*, especially the subtypes *stx2a* and *stx2d*, and the intimin-encoding gene *eae* (*E. coli* attaching and effacing), have been documented as factors associated with increased risk of HUS [3–6]. Classification of STEC has traditionally been based on seropathotypes, classifying serotypes according to association with severity of illness, outcome and outbreaks [7]. Knowledge of the evolution of pathogenic STEC has led to alternative classifications based on virulence factors, especially the *stx* genes, and their association with the development of HUS [4, 8].

In Norway, STEC infections have been mandatory notifiable since 1995 and reported via the Norwegian Surveillance System for Communicable Diseases (MSIS). Mandatory notification of diarrhoea-associated HUS was added to MSIS in 2006 following a national outbreak of STEC O103:H25 the same year [9, 10]. The National Reference Laboratory for Enteropathogenic Bacteria (NRL) at the Norwegian Institute of Public Health (NIPH) receives presumptive STEC isolates for verification and characterisation from all the Norwegian medical microbiological laboratories. Historically, laboratories have identified STEC by culturing, with focus on the identification of O157 [3, 11]. In the years following the 2006 outbreak, a majority of medical microbiological laboratories in Norway implemented PCR detection of *stx*. In recent years, multiplex PCR assays (in this paper referred to as “broad screening PCR”) have been introduced into routine primary diagnostics as a screening tool for gastrointestinal pathogens in some laboratories.

The number of notified STEC cases in Norway has increased in recent years, while the number of notified HUS cases has remained stable. This has challenged the existing system for infection control and follow-up of STEC cases. In 2016, the national guidelines on follow-up of STEC infections were revised in accordance with evidence on the association of HUS with STEC virulence factors [12]. Consequently, all STEC cases were assigned into either “high-virulent” or “low-virulent” STEC infection categories. To limit the socioeconomic consequences and the psychological impact of infection control measures for the patients and their families, only cases with high-virulent STEC infections identified in high-risk groups for disease transmission (e.g. food handlers, kindergarten children and staff) are now subject to follow-up.

The aim of this study was to investigate the observed increase of notified STEC cases in Norway from 2007 to 2017 in order to assess the effect of broad screening PCR implementation at the medical microbiological laboratories on the distribution and characteristics of notified STEC cases.

## Materials and methods

### Data collection

The notification criteria of STEC to MSIS are a clinically compatible case that is epidemiologically linked or is laboratory confirmed by (a) isolation of STEC positive for *stx1* or *stx2* gene(s), (b) detection of *stx1* or *stx2* gene(s) without isolation of strain, (c) detection of Stx in faeces without isolation of strain or (d) detection of STEC-specific antibodies in a HUS case. In absence of *stx*, a HUS patient with *eae*-positive *E. coli* and a patient with *eae-*positive *E. coli* with a known genotype (MLVA, multiple-locus variable-number of tandem repeat analysis), that has previously been identified in a HUS case, are also notifiable to MSIS. The latter is notified by the NRL as a probable case of STEC, which has lost its *stx* gene (STEC-LST).

We extracted data on all STEC cases notified to MSIS from 2007 to 2017 including demographics (age, sex, place), clinical presentation (symptoms, hospitalisation) and laboratory findings (date of sampling, diagnosing laboratory, serotype, *stx* subtype, presence of *eae* and *ehxA*, MLVA-type). Incomplete laboratory data in MSIS was supplemented with data from the NRL where available. In addition, we extracted data on all HUS (acute renal failure and at least microangiopathic haemolytic anaemia and/or thrombocytopenia) cases with an epidemiological link notified to MSIS in the same study period.

We gathered information on the implementation of broad screening PCR methodology at the medical microbiological laboratories from a national survey on laboratory practice from 2017, and through personal communication with the laboratories.

We extracted data on concomitant bacterial infections for all reported STEC cases from laboratories with broad screening PCR methodology. We defined a concomitant bacterial infection as notification of a pathogen included in the broad screening PCR panel (*Salmonella* spp., *Campylobacter* spp., *Shigella* spp., *Yersinia* spp. and/or other enteropathogenic *E. coli*) from the same laboratory and same sampling date as the STEC case.

### Categorisation of STEC cases

We categorised STEC cases into high- or low-virulent infections based on the 2016 revised guidelines [12].

A case was categorised as having a high-virulent STEC infection if

i) positive for *stx2* subtypes *2a, 2c*, *2d*, or
ii) positive for *stx1* subtype *1a* in a patient ≤ 5 years with bloody diarrhoea, or
iii) notified as a HUS patient, or
iv) negative for *stx*, but *eae*-positive *E. coli* strain (STEC-LST) with a genotype (MLVA-type) previously seen in a HUS case

A case was categorised as having a low-virulent STEC infection if

i) positive for *stx1* (not *1a* in a patient ≤ 5 years with bloody diarrhoea), or
ii) positive for *stx2* subtypes *2b*, *2e*, *2f*, *2g*

Cases that did not fulfil any of the above-mentioned criteria due to missing and/or insufficient data were categorised as having an unclassifiable STEC infection.

### Statistical analysis

We described cases in terms of demographic, clinical and microbiological characteristics. Incidence rates of notified STEC cases were calculated using population numbers provided by Statistics Norway registries (www.ssb.no).

We used chi-squared test for categorical variables to examine the distribution of demographics (sex, age, seasonality and place of infection), clinical (hospitalisation) and microbiological (serogroups and virulence profile) characteristics between cases with high-virulent and low-virulent STEC infections. We applied the Wilcoxon’s rank sum test to examine the differences between the two groups with respect to continuous variables (age).

We conducted time series analysis allowing for trends and seasonality (1 year periodicity) and calculated adjusted incidence rate ratios (aIRRs) with 95% confidence intervals (CIs) using negative binomial regression on 2007–2017 data for cases reported from laboratories that implemented broad screening PCR and from laboratories that did not implement this screening method.

We considered a *p* value of ≤ 0.05 as statistically significant. We performed all statistical analysis in Stata version 14 (Stata Corporation, College Station, Texas, USA).

## Results

### Notified STEC cases and categorisation of the cases

**Fig. 1:**
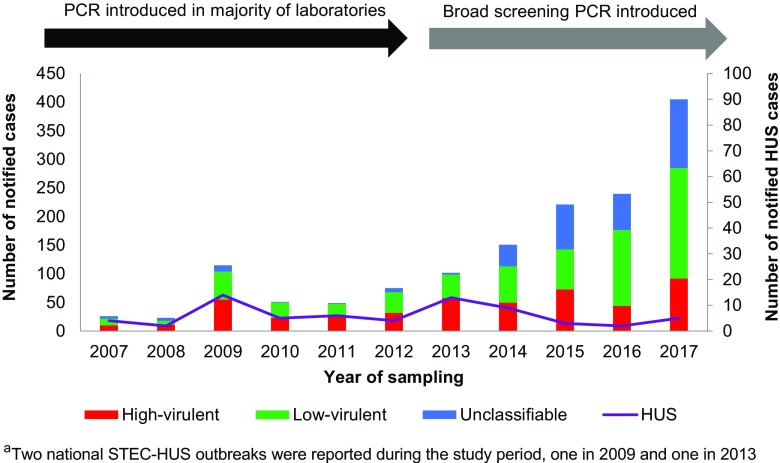
Annual distribution of cases categorised with high-virulent, low-virulent or unclassifiable Shiga toxin-producing *Escherichia coli* (STEC) infections notified to the Norwegian Surveillance System for Communicable Diseases (MSIS), 2007–2017 (*N* = 1458), and the number of HUS^a^ cases (purple line, *N* = 67). The time periods when the majority of clinical medical laboratories in Norway introduced PCR detection of *stx* and implemented broad screening PCR in five of the laboratories are indicated with a black and grey arrow, respectively

From 2007 to 2017, 1458 STEC cases were notified to MSIS. The median age of the cases was 21 years (range 0–97 years) and 51% of cases were female. The most frequent age group was ≤ 5 years with 37% of the cases. Among cases with known clinical outcome (1278), 5% developed HUS, 25% reported bloody diarrhoea as the worst clinical outcome and 11% were asymptomatic infections. At the time of notification, 26% of the cases were reported as hospitalised. For cases with available data on place of acquisition, 71% (902/1280) reported a domestically acquired infection. One or multiple *stx* subtype(s) was identified in 64% (936), *eae* in 55% (796) and *ehxA* in 48% (705) of all notified cases. The notified cases were categorised as 475 (33%) high-virulent, 652 (45%) low-virulent and 331 (23%) as unclassifiable STEC infections (Fig. 1).

The estimated annual STEC notification rate increased from 0.6 cases per 100,000 population in 2007 to 7.6 in 2017. In children (< 16 years of age), the estimated annual notification rate increased from 1.3 cases per 100,000 population in 2007 to 14.0 in 2017. In children ≤ 5 years of age, the estimated annual notification rate increased from 2.9 cases per 100,000 population in 2007 to 28.9 in 2017.

The NRL at NIPH received sample material (isolate or a mixed culture positive for *stx*) for 1135 (78%) of the notified cases including 99% of the cases with a high-virulent, 87% of the cases with a low-virulent and 30% of the cases with an unclassifiable STEC infection. The proportion of cases with sample material received decreased over the study period, from 96% (324/339) in the years 2007–2012 to 72% (811/1119) in 2013–2017. The lowest yearly proportion was recorded in 2017 (64%, 260/405).

### Comparing cases with high-virulent versus low-virulent STEC infection

#### Demographic and clinical data

We observed a difference in the age distribution between cases with high-virulent and low-virulent STEC infections, with an estimated median age of 5 years (range 0–97 years) compared to 22 years (range 0–93 years) respectively in the two groups (*p* < 0.001). Furthermore, we identified a difference in seasonality between the two groups with a higher proportion of cases with high-virulent STEC infections during summer (36% vs 29%) and less during winter (14% vs 21%) (*p* = 0.008). No difference was observed between the two groups in terms of distribution of sex or place of infection (domestically acquired or infected abroad). Cases with high-virulent STEC infection were more frequently reported as hospitalised than cases with a low-virulent infection (42% vs 21%, *p* < 0.001) (Table 1).

**Table 1:** Demographic and clinical characteristics of cases categorised with high-virulent (number of cases; *N* = 475) versus low-virulent (*N* = 652) Shiga toxin-producing *Escherichia coli* (STEC) infections, notified to the Norwegian Surveillance System for Communicable Diseases (MSIS), Norway, 2007–2017 (*N* = 1127)

Variable	Category	All cases^a^ (*N* = 1127)		High-virulent STEC cases^a^ (*N* = 475)		Low-virulent STEC cases^a^ (*N* = 652)		Chi-square *p* value^b^
		*N*	%	*N*	%	*N*	%	
Sex	Female	585	52	258	54	327	50	0.167
	Male	542	48	217	46	325	50	
Age group	≤ 5	458	41	242	51	216	33	< *0.001*
	6–15	101	9	39	8	62	9	
	16–41	295	26	108	23	187	29	
	42–64	163	14	48	10	115	18	
	65+	110	10	38	8	72	11	
Seasonality	Winter	204	18	66	14	138	21	*0.008*
	Spring	168	15	71	15	97	15	
	Summer	360	32	170	36	190	29	
	Autumn	395	35	168	35	227	35	
Reported infected abroad	No	747	74	332	76	415	73	0.255
	Yes	260	26	105	24	155	27	
Hospitalised	No	750	71	260	59	490	79	< *0.001*
	Yes	311	29	182	41	129	21	

^a^The numbers and proportions reported per column for each characteristic use the number of cases with available (known) information regarding each characteristic

^b^A *p* value of ≤ 0.05 (italicised) was considered statistically significant

#### Microbiological characteristics

Subtype of *stx* was available for 85% (403/475) of the cases with high-virulent STEC and for 82% (532/652) of the cases with low-virulent STEC infections. In the former group, the most commonly identified subtypes were *stx2a* (224/403; 56%) and *Stx2c* (157/403; 39%), whereas *stx1a* (278/532; 52%) and *stx2b* (159/532; 30%) were more frequently seen in the low-virulent group (*Online Resource 1*). Furthermore, serogroups O157 (43% vs 1%), O145 (15% vs 5%) and O26 (17% vs 9%) were more commonly identified in cases with high-virulent STEC infection than in the low-virulent group, while the opposite was observed for serogroup O103 (4% vs 23%) (*p* < 0.001). Additionally, virulence genes *eae* and *ehxA* were more prevalent in the high-virulent group (87% vs 51%, *p* < 0.001 and 77% vs 51%, *p* < 0.001, respectively) (Table 2).

**Table 2:** Microbiological characteristics of cases categorised with high-virulent (number of cases; *N* = 475) versus low-virulent (*N* = 652) Shiga toxin-producing *Escherichia coli* (STEC) infections, notified to the Norwegian Surveillance System for Communicable Diseases (MSIS), Norway, 2007–2017 (*N* = 1127)

Characteristics	Category	All cases^a^ (*N* = 1127)		High-virulent STEC cases^a^ (*N* = 475)		Low-virulent STEC cases^a^ (*N* = 652)		Chi-square *p* value^b^
		*N*	%	*N*	%	*N*	%	
Serogroup distribution	O157	195	20	192	43	3	1	< *0.001*
	O103	136	14	16	4	120	23	
	O26	126	13	78	17	48	9	
	O145	96	10	67	15	29	5	
	Other	418	43	96	21	322	62	
*eae* ^c^	Positive	710	67	406	87	304	51	< *0.001*
	Negative	352	33	60	13	292	49	
*ehxA* ^d^	Identified	700	62	365	77	335	51	< *0.001*
	Not identified	427	38	110	23	317	49	

^a^The numbers and proportions reported per column for each characteristic use the number of cases with available (known) information regarding each characteristic

^b^A *p* value of ≤ 0.05 (italicised) was considered statistically significant

^c^Intimin-encoding gene (*Escherichia coli* attaching and effacing)

^d^Enterohaemolysin-encoding gene (enterohaemolysin)

### Implementation of broad screening PCR

Five medical microbiological laboratories implemented broad screening PCR during the study period. The different laboratories implemented broad screening PCR on the following dates: November 1st 2013, June 1st 2014, March 16th 2015, August 4th 2015 and April 1st 2017. The second laboratory had no record of notified STEC cases prior to 2013 and was therefore excluded from the time series analysis. The remaining 17 medical microbiological laboratories in Norway did not implement broad screening PCR during the study period.

**Fig. 2:**
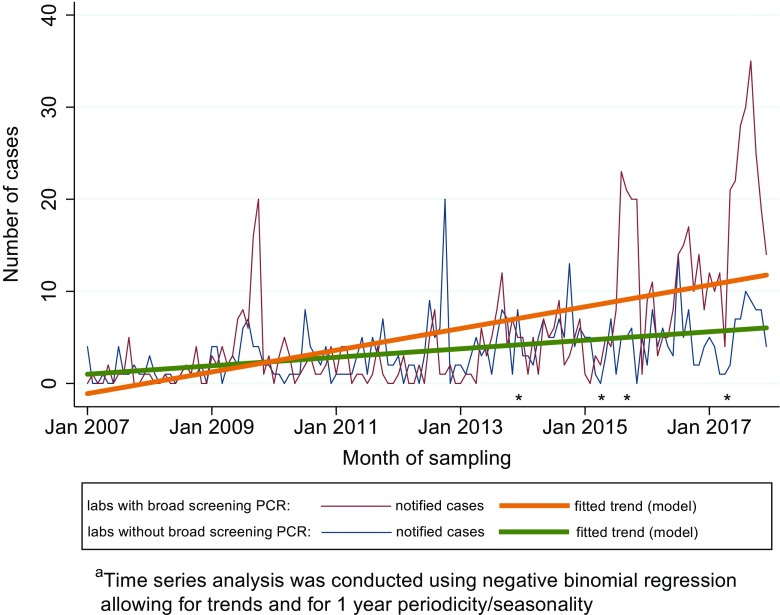
Monthly distribution of notified Shiga toxin-producing *Escherichia coli* (STEC) cases with fitted trend based on time series analysis model^a^ for the four medical microbiological laboratories that implemented broad screening PCR (*N* = 728 cases) and for the 17 laboratories that did not implement broad screening PCR (*N* = 461 cases), Norway, 2007–2017. The different time points that the four laboratories started implementing broad screening PCR are marked with an asterisk (*)

Adjusted for 1-year periodicity (significant in both models; sine-wave *p* < 0.001, cosine-wave *p* < 0.001), we observed a higher increasing monthly trend in STEC cases (aIRR = 1.020; 95% CI 1.016–1.024) notified from the four laboratories that had implemented broad screening PCR, compared to laboratories that had not implement this method (aIRR = 1.011; 95% CI 1.007–1.014, non-overlapping confidence intervals) (Fig. 2).

**Fig. 3:**
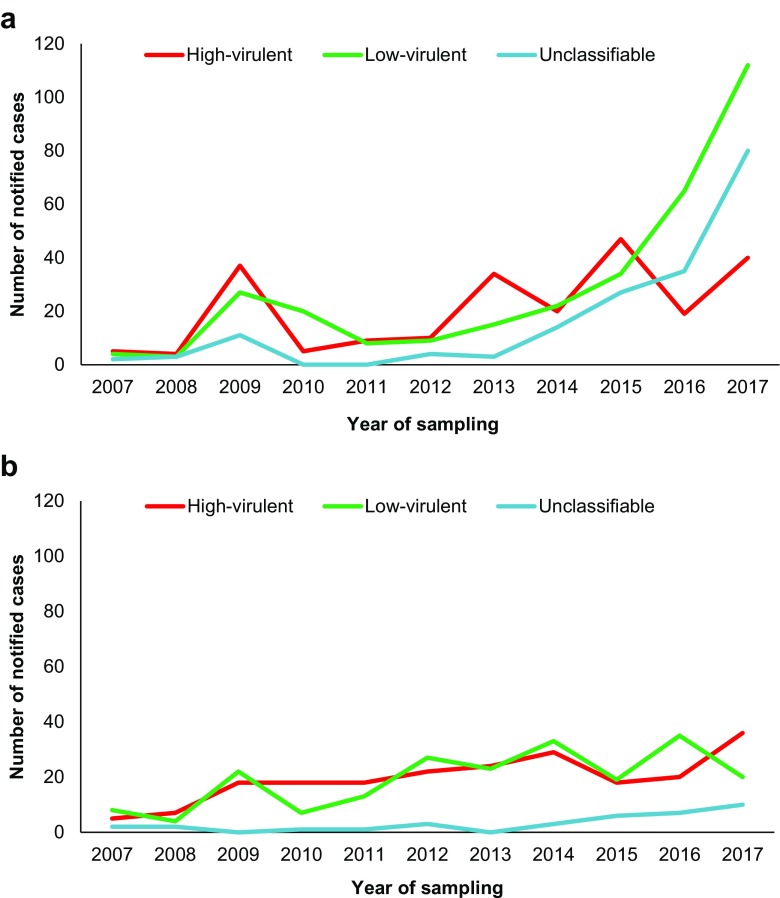
Annual distribution of cases notified and categorised with high-virulent, low-virulent or unclassifiable Shiga toxin-producing *Escherichia coli* (STEC) infections from **a** four laboratories that implemented broad screening PCR (*N* = 728) and **b** 17 laboratories that did not (*N* = 461), Norway, 2007–2017

The difference in annual number of cases categorised as high-virulent, low-virulent or unclassifiable STEC infections was assessed in laboratories with and without broad screening PCR (Fig. 3). Throughout the study period, laboratories that implemented broad screening PCR notified 260 (26%) cases with high-virulent, 441 (44%) cases with low-virulent and 296 (30%) cases with unclassifiable STEC infections, while the remaining laboratories notified 215 (47%), 211 (46%) and 35 (8%) cases in the different categories, respectively (*Online Resource 2*). From 2013, when the first laboratory introduced broad screening PCR, there was an increase in the proportion of cases with low-virulent and unclassifiable STEC infections notified from these laboratories. In the laboratories without broad screening PCR, the distribution of categorised cases was relatively stable throughout the study period. In 2017, the proportion of cases with high-virulent STEC infection was 17% in cases reported from laboratories with broad screening PCR, and 55% in cases reported from laboratories without broad screening PCR. Before 2013, both groups of laboratories had comparable distribution of notified cases categorised as high-virulent, low-virulent or unclassifiable STEC infections.

### Concomitant bacterial infections

During the study period, 997 STEC cases were notified from the five laboratories that had implemented broad screening PCR. For 115 (12%) of these cases, one or more concomitant enteropathogenic bacteria were identified. An increasing proportion of cases with concomitant bacteria was observed after the introduction of broad screening PCR (15%) compared to before (7%). In 2017, concomitant bacteria were detected in 15% (51/339) of all notified STEC cases, which were 44% of all concomitant bacteria identified during the study period. After the implementation of broad screening PCR, concomitant bacteria were identified in 11 (9%) cases with high-virulent, 26 (8%) cases with low-virulent and 59 (23%) cases with unclassifiable STEC infections. The most common groups of concomitant bacteria were *Campylobacter* spp. (37%, 43/115 cases), followed by atypical enteropathogenic *Escherichia coli* (aEPEC) (31%, 36/115), and *Salmonella* spp. (12%, 14/115).

## Discussion

In this study, we have investigated the increase of notification of STEC cases to MSIS in Norway from 2007 to 2017. We observed an overall increase throughout the study period, with annual number of STEC notifications relatively stable until 2014, followed by a sharp increase in the following years. A similar observation was seen in our neighbouring countries, Sweden and Denmark [13, 14]. Furthermore, an overall steady increase of notified STEC cases has been reported in Europe and the US over several years [15, 16], mainly due to identification of non-O157 STEC infections [16, 17]. The 2016 notification rate in Norway (4.6 per 100,000 population) was the fourth highest reported in the European Union summary report, following Ireland, Sweden and Switzerland (15.6, 6.5 and 5.5, respectively) [15].

The recent upsurge in Norway coincided with the implementation of broad screening PCR for enteropathogenic bacteria at five of the larger medical microbiological laboratories in the country. This was prominent in cases categorised with a low-virulent STEC infection. In 2016 and 2017, the number of notified cases with low-virulent STEC was more than double than that of high-virulent STEC infections, largely attributed to cases notified from laboratories that had implemented broad screening PCR during the study period. Meanwhile, the annual distribution of cases categorised as high- or low-virulent STEC infections was quite stable during the study period in the remaining 17 laboratories. In addition, the annual number of notified HUS cases remained stable. HUS surveillance can be used to monitor STEC occurrence [15, 18, 19]. Based on this, a previous study on paediatric HUS and STEC in Norway strongly suggested an underestimation of STEC incidence [20]. From this, one would expect an increased detection rate of low-virulent STEC in Norwegian laboratories when implementing unselected screening, as seen in our study. Other reports have shown similar effect; a study from Denmark noted an 88% increase of STEC in an associated laboratory after implementation of non-selective stool screening [17]. This likely reflects both the effect of a broader diagnostic approach and improved detection for non-O157 STEC over the last decade [16, 17, 21]. A moderate increase of STEC cases notified to MSIS was also observed in laboratories without broad screening PCR diagnostics, probably due to implementation of selective PCR methods for detection of STEC following the 2006 outbreak [10, 22].

Furthermore, we observed a marked increase in notified cases without identification of toxin subtype, which is also reflected by trends reported in the European surveillance data [15]. The majority of these cases were categorised with unclassifiable STEC infections in our study. Mostly, these were *stx1/2* positive and culture negative, which is a common finding in culture-independent (PCR-based) STEC detection [23]. Such cases are often associated with high *stx* C_T_ values, which may suggest the presence of non-viable STEC [8], or represent identification of *stx* from free temperate bacteriophages [23]. Other bacteria, such as *Escherichia albertii* and *Citrobacter freundii*, may also carry *stx* [24, 25]. Furthermore, the unclassifiable STEC infections were highly represented in cases where concomitant enteropathogenic bacteria had been notified, predominantly in laboratories that had implemented broad screening PCR. Studies have shown that concomitant enteropathogens occur frequently in *stx*-positive samples compared to samples with other common enteropathogens [8, 26]. These are all potential sources of notified *stx* findings that may not yield positive cultures, and likely contribute to the increase of cases with unclassified STEC infections when non-selective screening is applied. In addition, the prevalence of STEC or *stx* in healthy carriers is mostly unknown, but important to consider when evaluating the clinical impact of a *stx*-positive finding. A recent study reported an incidence rate of STEC infection in asymptomatic adults as high as 84.2 per 100,000 population [27]. Interestingly, many of these STEC belonged to O serogroups that were untypeable or rarely found in symptomatic patients and > 80% were *eae* negative. The *stx*-positive but culture-negative STEC cases pose a growing challenge to the STEC surveillance system, as no cultures are available to the national reference laboratories for molecular characterisation and cluster detection. In Norway, most of these cases would require to be followed up as a probable high-virulent STEC infection until three consecutive stool samples are negative or a positive culture can confirm a low-virulent STEC [12]. Consequently, there is an ongoing debate regarding the increasing workload related to cases with unclassifiable STEC infections. As the differentiation of STEC is predominantly based on *stx* subtype, standardised subtyping directly from DNA obtained from enriched broth from positive samples rather than STEC isolates could improve subtype determination rates regardless of culture yield [8]. More specific methods, such as microfiltration of samples, have also been suggested to avoid interference of free bacteriophages in STEC identification [23]. Regardless, studies assessing the clinical relevance of such cases are needed.

Broad screening PCR provide fast and sensitive identification and allow for rapid exclusion of possible enteropathogens [26, 28]. However, laboratories using broad screening PCR methodology test stool samples against a panel of common enteropathogenic bacteria instead of a selective diagnostic approach based on clinical assessment. Consequentially, they contribute to higher identification rates of both primary enteropathogens and concomitant bacteria [26, 28, 29]. Higher identification rates contribute to an increased socioeconomic burden for public health services, those directly affected, and the society [8, 9, 30]. According to our findings, a large proportion of STEC infections can effectively be categorised as low-virulent, thus largely decreasing the number of cases in need of strict follow-up and control measures. We consider this an important and necessary response to the constant improvement of STEC detection methods. Others have suggested more drastic measures, such as reserving multiplexed panels to specific patient populations to improve test utilisation [28].

There are multiple broad screening PCRs commercially available and medical microbiological laboratories in Norway are autonomous in their choice of diagnostic methodology for STEC infections. This can lead to variability in the capacity to detect different STEC between laboratories as the methodologies differ in sensitivity. In addition, the laboratories are not required to inform the NRL of any changes in diagnostic methodologies, including implementation of broad screening PCR. While the increase of STEC cases following the introduction of broad screening PCR can be observed in the number of notified cases, the exact date of implementation was unknown for one laboratory. Although we contacted the laboratory to confirm the date of introduction, it could only provide the month of implementation. This may have resulted in minimal errors in the grouping of cases pre- or post-introduction of broad screening PCR.

## Conclusions

The increase in notified STEC cases in Norway from 2007 to 2017 is largely attributable to implementation of broad screening PCR at five of the larger medical microbiological laboratories in the country. The increase was prominent in cases categorised with a low-virulent STEC infection. We recommend NIPH to maintain differentiated control measures for STEC cases to avoid follow-up of low-virulent STEC infections. We recommend microbiological laboratories in Norway to consider a more cost-effective broad screening PCR strategy that enables differentiation of high-virulent STEC infections.

## Electronic supplementary material

ESM 1 (DOCX 29 kb)

## Abbreviations

*aEPEC*: Atypical enteropathogenic *Escherichia coli*
*E. coli*: *Escherichia coli*
*eae*: *E. coli* attaching and effacing
*ehxA*: Enterohaemolysin
*HUS*: Haemolytic-uraemic syndrome
*MLVA*: Multiple-locus variable-number of tandem repeat analysis
*MSIS*: The National Surveillance System for Communicable Diseases
*NIPH*: Norwegian Institute of Public Health
*NRL*: National Reference Laboratory for Enteropathogenic Bacteria
*NSF*: Non-sorbitol fermenting
*SF*: Sorbitol fermenting
*Stx*: Shiga toxin
*STEC*: Shiga toxin-producing *E. coli*
*STEC-LST*: STEC who have lost their toxin(s)
*VTEC*: Verocytotoxigenic *E. coli*
