# Supplementary material for: Implementation of multiplex PCR diagnostics for gastrointestinal pathogens linked to increase of notified Shiga toxin-producing Escherichia coli cases in Norway, 2007–2017
Source: Eur J Clin Microbiol Infect Dis. 2019 Jan 24;38(4):801–9. doi: 10.1007/s10096-019-03475-5 (PMC6424946; doi:10.1007/s10096-019-03475-5)
Supplement: Supplementary file 1 — (DOCX 29 kb) [file 10096_2019_3475_MOESM1_ESM.docx]

**Implementation of multiplex PCR diagnostics for gastrointestinal pathogens linked to increase of notified Shiga toxin-producing *Escherichia coli* cases in Norway, 2007-2017.**

**Gaute Reier Jenssen^1^**^,2,3§^, **Lamprini Veneti**^1^, **Heidi Lange**^1^, **Line Vold**^1^, **Umaer Naseer^1^**, **Lin T. Brandal**^1^

^1^Department of Infectious Disease Epidemiology, Norwegian Institute of Public Health, Oslo, Norway

^2^Faculty of Medicine, University of Oslo, Oslo, Norway

^3^Oslo University Hospital, Oslo, Norway

^§^Corresponding author: Gaute Reier Jenssen, Department of Infectious Disease Epidemiology, Norwegian Institute of Public Health, Postboks 4404 Nydalen, NO-0403 Oslo, Norway. Tel +47 21077000. E-mail: [g.r.jenssen@medisin.uio.no](mailto:g.r.jenssen@medisin.uio.no)

**Online Resource 1** Distribution of *stx* subtypes in Shiga toxin-producing *Escherichia coli* (STEC) from cases categorised with high-virulent (N = 403) and low-virulent (N = 532) STEC infections, notified to the Norwegian Surveillance System for Communicable Diseases (MSIS), Norway, 2007-2017 (N = 935)

|  |  | **All cases^b^**  **(N=935)** |  | **High-virulent**  **STEC cases^b^**  **(N=403)** |  | **Low-virulent**  **STEC cases^b^**  **(N=532)** |  |
| --- | --- | --- | --- | --- | --- | --- | --- |
| **Variable** | Category | No | % | No | % | No | % |
| ***stx1*^a^** | *Stx1a* | 421 | 78% | 143 | 99% | 278 | 70% |
|  | *Stx1c* | 111 | 21% | 1 | 1% | 110 | 28% |
|  | *Stx1d* | 6 | 1% | 0 | 0% | 6 | 2% |
| ***stx2*^a^** | *Stx2a* | 180 | 31% | 180 | 48% | 0 | 0% |
|  | *Stx2a+b* | 2 | <1% | 2 | <1% | 0 | 0% |
|  | *Stx2a+c* | 33 | 6% | 33 | 9% | 0 | 0% |
|  | *Stx2a+d* | 8 | 1% | 8 | 2% | 0 | 0% |
|  | *Stx2a+g* | 1 | <1% | 1 | <1% | 0 | 0% |
|  | *Stx2b* | 160 | 27% | 1 | <1% | 159 | 75% |
|  | *Stx2c* | 124 | 21% | 124 | 33% | 0 | 0% |
|  | *Stx2d* | 25 | 4% | 25 | 7% | 0 | 0% |
|  | *Stx2e* | 9 | 2% | 0 | 0% | 9 | 4% |
|  | *Stx2f* | 37 | 6% | 0 | 0% | 37 | 18% |
|  | *Stx2g* | 7 | 1% | 0 | 0% | 7 | 3% |

^a^Including 189 cases were both *stx1* and *stx2* were identified (115 high-virulent STEC, 74 low-virulent STEC).

^b^The numbers and proportions reported per column for each characteristic use the number of cases with available (known) information regarding each characteristic.

**Online Resource 2** Distribution of Shiga-toxin producing *Escherichia coli* (STEC) cases categorised as high-virulent (H), low-virulent (L) and unclassifiable (U) infections, notified to the Norwegian Surveillance System for Communicable Diseases (MSIS), Norway, 2007-2017; A) from medical microbiological laboratories (Lab.) with broad screening PCR (N = 5, presented individually) and B) from laboratories without (N = 17). Numbers in bold designate years (2009 and 2013) with national STEC-HUS outbreaks. Grey areas designate years where the laboratory implemented broad screening PCR

|  | **A) Broad screening PCR implemented in study period** | | | | | | | | | | | | | | | | | | **B) Other** | | |
| --- | --- | --- | --- | --- | --- | --- | --- | --- | --- | --- | --- | --- | --- | --- | --- | --- | --- | --- | --- | --- | --- |
| **Lab.** | Lab. 1 | | | Lab. 2 | | | Lab. 3 | | | Lab. 4 | | | Lab. 5 | | | All | | | All | | |
| **Year** | H | L | U | H | L | U | H | L | U | H | L | U | H | L | U | H | L | U | H | L | U |
| 2007 | 0 | 0 | 0 | 0 | 0 | 0 | 0 | 0 | 0 | 3 | 3 | 0 | 2 | 1 | 2 | 5 | 4 | 2 | 5 | 8 | 2 |
| 2008 | 0 | 0 | 0 | 0 | 0 | 0 | 0 | 0 | 0 | 2 | 2 | 2 | 2 | 1 | 1 | 4 | 3 | 3 | 7 | 4 | 2 |
| **2009** | **3** | **1** | **0** | **0** | **0** | **0** | **1** | **0** | **0** | **24** | **19** | **10** | **9** | **7** | **1** | **37** | **27** | **12** | **18** | **22** | **0** |
| 2010 | 1 | 6 | 0 | 0 | 0 | 0 | 0 | 0 | 0 | 4 | 13 | 0 | 0 | 1 | 0 | 5 | 20 | 0 | 18 | 7 | 1 |
| 2011 | 2 | 2 | 0 | 0 | 0 | 0 | 0 | 0 | 0 | 7 | 3 | 0 | 0 | 3 | 0 | 9 | 8 | 0 | 18 | 13 | 1 |
| 2012 | 1 | 1 | 0 | 0 | 0 | 0 | 1 | 1 | 0 | 6 | 4 | 3 | 2 | 3 | 1 | 10 | 9 | 4 | 22 | 27 | 3 |
| **2013** | **2** | **2** | **0** | **0** | **3** | **0** | **1** | **0** | **0** | **26** | **10** | **3** | **5** | **3** | **0** | **34** | **18** | **3** | **24** | **23** | **0** |
| 2014 | 5 | 10 | 3 | 1 | 8 | 21 | 0 | 0 | 4 | 11 | 9 | 7 | 4 | 3 | 0 | 21 | 30 | 35 | 29 | 33 | 3 |
| 2015 | 9 | 9 | 12 | 8 | 17 | 45 | 4 | 11 | 11 | 25 | 10 | 4 | 9 | 4 | 0 | 55 | 51 | 72 | 18 | 19 | 6 |
| 2016 | 6 | 14 | 16 | 5 | 33 | 21 | 5 | 16 | 6 | 6 | 32 | 13 | 2 | 3 | 0 | 24 | 98 | 56 | 20 | 35 | 7 |
| 2017 | 6 | 13 | 13 | 16 | 61 | 30 | 6 | 28 | 16 | 14 | 31 | 9 | 14 | 40 | 42 | 56 | 173 | 110 | 36 | 20 | 10 |
| **Total** | 35 | 58 | 44 | 30 | 122 | 117 | 18 | 56 | 37 | 128 | 136 | 51 | 49 | 69 | 47 | 260 | 441 | 296 | 215 | 211 | 35 |
